# Psychometric Properties of the Persian Translation of Video Gaming Addiction Test

**DOI:** 10.32598/bcn.9.10.345

**Published:** 2019-09-01

**Authors:** Zahra Sadat Hosseini, Roya Delpazirian, Hossein Mohajeri, Peyman Hassani Abharian

**Affiliations:** 1. Institute for Cognitive Sciences Studies (ICSS), Tehran, Iran.; 2. Brain and Cogntive Clinic, Insitute for Cognitive Sciences Studies (ICSS), Tehran, Iran.; 3. Psychology Department, Faculty of Psychology and Educational Sciences, Allameh Tabataba’i University, Tehran, Iran.

**Keywords:** Video gaming Addiction test, Reliability, Validity, Psychometric properties

## Abstract

**Introduction:**

The current study aims to measure the validity, reliability, and psychometric properties of the Persian translation of the Video Gaming Addiction Test (VAT).

**Methods:**

A total of 280 young men (14–20 years old) (Mean±SD age: 17.22±1.8 years), including excessive gamers and normal subjects, entered the study. They answered VAT, Visual Analog Scale (VAS), and Conner-Davidson Resilience Scale (CD-RISC). The VAT was translated and then back-translated. For testing reliability, we used the Cronbach alpha, split-half method, and Guttman method. Also, convergent and discriminant validity were tested to examine the construct validity of the translated version of VAT.

**Results:**

The Cronbach alpha for the total scale was 0.81. Also, after splitting questions in half, the Cronbach alpha values for these halves were 0.71 and 0.69. Six Guttman lambdas were calculated with 0.75 minimum and 0.82 maximum, all showing good reliability of the test. Convergent validity was tested by testing the correlation between VAT and VAS. The Pearson correlation was fond 0.73, showing a strong relationship between the two factors (P<0.001). For testing discriminant validity, the association of VAT with CD-RISC was tested, showing no correlation between these scales (r=−0.157; P=0.09).

**Conclusion:**

The Persian translation of the VAT is valid and reliable, and it is appropriate for research and clinical use with acceptable properties, similar to the original version.

## Highlights

- Video Gaming Addiction Test is a reliable and valid tool for assessing Internet gaming disorder mentioned in DSM-5.
- Split-Half technique and Cronbach’s Alpha method were used to estimate the reliability of the Persian version of Video Gaming Addiction Test.
- The Persian version of the Video Gaming Addiction Test is valid and reliable, and it is appropriate for using in clinical studies.

## Plain Language Summary

Internet gaming disorder was mentioned in the new version of the Diagnostic and Statistical Manual for Mental Disorders (DSM-5). It is described as the “Persistent and recurrent use of the Internet to engage in games, often with other players, leading to clinically significant impairment or distress as indicated by 5 (or more) of the following in a 12-month period”. The diagnostic criteria are withdrawal symptoms; preoccupation with Internet games; tolerance (i.e. increase in devoting time for gaming); Ineffective efforts to manage it; lack of appeal in other hobbies; inability to control the behavior; deception of others about spending hours on gaming; the use of Internet games to improve mood, an important relationship harm, loss of job, career, or educational opportunity, or other important matters. Among the instruments measuring different characteristics of Internet gaming disorder, only Video Gaming Addiction Test (VAT) covers all aspects of this disorder mentioned in DSM-5. In this study, the psychometric characteristics of the Persian version of VAT were investigated to be used on Iranian population. Split-Half technique and Cronbach’s Alpha method were used to estimate the reliability of this Test and convergent and discriminant validity were tested to examine its construct validity. Results showed that the Persian version of VAT is also valid and reliable, and it is appropriate for using in clinical studies.

## 1. Introduction

Since the first report of the Internet gaming addiction problem published 1983, the proper identification of problems involving this behavior was vexed among clinicians. The properties and components of this disorder were discussed in many research studies (Griffiths M., 2005; Griffiths, Kuss, & King, 2012). After about 30 years, the American Psychiatric Association documented the Internet Gaming Disorder officially as one of the behaviors that might be considered by researchers and clinicians. Internet gaming disorder was mentioned in the new version of the Diagnostic and Statistical Manual for Mental Disorders (DSM-5) appendix for the first time in June 2013 (American Psychiatric Association, 2013).

Internet gaming disorder is described as the “Persistent and recurrent use of the Internet to engage in games, often with other players, leading to clinically significant impairment or distress as indicated by five (or more) of the following in a 12-month period”, stated by the new DSM-5 framework (American Psychiatric Association, 2013). The diagnostic criteria are withdrawal symptoms; preoccupation with Internet games; tolerance (i.e. increase in devoting time for gaming); ineffective efforts to manage it; lack of appeal in other hobbies; inability to control the behavior; deception of others about spending hours on gaming; the use of Internet games to improve mood, an important relationship harm, loss of job, career, or educational opportunity, or other important matters (American Psychiatric Association, 2013).

So far, researchers have developed multiple psychometric instruments, each of which measuring different characteristics of Internet gaming disorder (Charlton & Danforth, 2007; Kim & Kim, 2010; King, Delfabbro, & Zajac, 2011; Salguero & Moran, 2002; van Rooij, Schoenmakers, van den Eijnden, Vermulst, & van de Mheen, 2012; Demetrovics, et al., 2012; Gentile, 2009; Choo, et al., 2010; Lemmens, Valkenburg, & Peter, 2009). Among these instruments, only VAT covers all aspects of this disorder mentioned by DSM-5 (van Rooij et al. 2012).

The aim of Video Game Addiction Test (VAT), a revised version of the Compulsive Internet Use Scale (CIUS) with 14 items in a Likert-type 5-point scale (0 for never; 1 for seldom; 2 for sometimes; 3 for often; and 4 for very often), is to assess the level of addiction of video game, so that higher scores indicate a higher level of the addiction symptom. To test its validity and reliability, Van Rooij used a sample comprising 10 Dutch secondary schools. The overall sample response rate was 83%, leading to 4074 completed questionnaires. Adolescents items that did not play games at all were removed from the dataset (n=1024). For scale validation, they excluded the questionnaires having below 4 completed items on the VAT (n=156).

This screening made the final dataset, including 2894 cases, with a total average age of 14.3 (SD=1.0) years. The test reliability was checked by Confirmatory Factor Analysis and measurement of variance. The participants also filled CIUS (Meerkerk, Van Den Eijnden, Vermulst, & Garretsen, 2009), game addiction scale (Lemmens et al. 2009), Rosenberg’s negative self-esteem scale (Rosenberg, 1965), the UCLA loneliness scale (Russell, Peplau, & Ferguson, 1978), the depressive mood list (Kandel & Davies, 1982). Also, the revised social anxiety scale for children (La Greca & Stone, 1993) used for testing convergent and discriminant validity (van Rooij et al. 2012).

Finally, the psychometric characteristics of VAT were shown to be very good, enjoying the benefit of having a strong relationship with the CIUS, a current Internet addiction scale; scores were proven to be trustworthy in the diagnosis of Internet gaming addiction (van Rooij et al. 2012) Materials and Methods

### Study sample

A total of 300 young men with the Mean±SD age of 17.22±1.8 (range 14–20 years), including normal subjects (from 3 high schools) and excessive gamers (from 2 gaming clubs), entered the study. To validate the scale, the participants with less than four completed items on each survey were excluded (n=20). Finally, 280 cases remained with dataset. All participants provided written consent and signed an agreement before participating in the study.

### Measures

Persian translated version of VAT: As discussed before, the reliability and validity of VAT were already tested (van Rooij et al. 2012) and we used a translated version of the questionnaire. Two translators did the translation and back-translation. One of them did not know the original English text. The final translation was modified with consensus.

VAS: The 3-item version of VAS was used to measure the level of dependency on gaming (with its Persian phrasing adjusted) and included these questions:

1. How much do you want to play a video game now?; 2. How much do you need to play a video game now?; 3. How much are you forced to play a video game now? The participants answered these questions by rating their tendency from 0 to 10.

Persian translated version of CD-RISC: The CD-RISC was created to improve the current values of resilience. It is a self-administered questionnaire with 25 items evaluated based on a 5-point Likert-type scale from 0 to 4; 4 for “True nearly all the time”, 3 for “Often true”, 2 for “Sometimes true”, 1 for “Rarely true”, and 0 for “Not true at all” exhibiting good psychometric properties (Connor & Davidson, 2003). We used the Persian version of the questionnaire, whose reliability and validity were already tested by Besharat (Besharat, 2007).

## 2. Methods

Reliability was evaluated with three methods. Firstly, internal consistency evaluated, which is the consistency of people’s responses across the items on a multiple-item measure. The internal consistency was verified with the Cronbach alpha coefficients, as well as using Guttman split-half method. Split-half is a way of measuring consistency, where a test is divided into 2 parts, and the scores of each half of the test are compared with the other half. The Cronbach alpha value was calculated separately for questions 1 to 7 and 8 to 14, and the correlation of these two groups was examined.

We also used the Guttman lambda-6 method to test reliability. Guttman (Guttman, 1945) proposed a series of 6 so-called lambda indices to assess a similar lower bound for reliability; Guttman lambda-3, the lowest bound, was strictly equivalent to the Cronbach alpha. Instead of estimating the actual variance of every item as the average covariance between items, we considered the amount of variance in each item that could be accounted for by the linear regression of all other items (aka, the squared multiple correlations). We got the lambda-6 estimate, which might be computed for multi-scale instrument, as well.

We evaluated the construct validity (divergent or discriminant and convergent) by examining the correlation of the index subscales scores with the other values used in the survey (VAS for convergent and CD-RISC for discriminant). Converging subscale with the scores of instruments targeting the same construct and diverging from the scores given by instruments or scales evaluating a different one can be expected. We used the Pearson correlation to measure these relationships.

Secondly, factor analysis was used to determine whether or not the VAT had factor validity. To evaluate the questionnaire’s factor validity and fitting, we used the exploratory factor analysis and confirmatory factor analysis using SPSS. To determine how many significant factors saturates VAT, we inspected three parameters:

1. Eigenvalues; 2. Assigned variance ratio of each factor; 3. Eigenvalues plot (screen test).

## 3. Results

### Reliability

The calculated Cronbach alpha value for total items was 0.81, meaning that the instrument has a good internal consistency. Also, Table 1 presents the obtained values of Guttman split-half. The minimum value of halves and total is for the second half with the value of 0.69, which indicates a good consistency for the questionnaire.

**Table 1.** The Guttman split-half calculated values

**Reliability Method**	**First Half**	**Second Half**	**Total**
Cronbach alpha	0.708	0.687	-
Guttman split-half coefficient	-	-	0.768

Table 2 presents the Guttman lambda values. The minimum value was 0.752 for lambda-1, indicating good consistency in the instrument.

**Table 2.** The Guttman lambda values

**Reliability Method**	**λ1**	**λ2**	**λ3**	**λ4**	**λ5**	**λ6**
Guttman lambda values	0.752	0.814	0.810	0.768	0.791	0.817

### Validity

The Pearson correlation coefficient was calculated to determine the correlation between the Visual Analog Scale and Video Addiction test and between Conner-Davidson Resilience Scale and Video Addiction test. This coefficient with P<0.001 was 0.726, indicating a significant correlation between VAS and VAT, and VAT has convergent validity.

The correlation between CD-RISC and VAT was tested, and no association was found between these tests (r=−0.157; P=0.09). This result indicates the discriminant validity of the translated instrument.

Table 3 presents the essential statistical data of VAT’s factor analysis. Eigenvalues of the first, second, and third factors are over 1. These factors altogether have 51.17% of total questions variance.

**Table 3.** VAT factor analysis statistical values

**Question**	**Initial**	**Factor**	**Eigenvalue**	**Variance Explained %**	**Cumulative Variance %**
1	1	1	4.90	35.05	35.05
2	1	2	1.22	8.73	43.78
3	1	3	1.03	7.38	51.17
4	1	4	0.92	6.65	57.74
5	1	5	0.89	6.38	64.12
6	1	6	0.75	5.35	69.48
7	1	7	0.70	5.05	74.53
8	1	8	0.67	4.78	79.31
9	1	9	0.60	4.30	83.62
10	1	10	0.53	3.81	87.43
11	1	11	0.48	3.49	90.92
12	1	12	0.45	3.23	94.16
13	1	13	0.41	2.98	97.14
14	1	14	0.40	2.85	100

The extracted factors were transferred to the new axis by rotating the varimax to find a more explicit analysis. Table 4 presents the results of the factor loadings of the questionnaire’s varimax rotation.

**Table 4.** VAT questions rotated factor matrix

**Question**	**The First Factor**	**The Second Factor**	**The Third Factor**
1	0.158	0.364	0.680
2	0.045	−0.120	0.736
3	0.093	0.662	0.332
4	0.170	0.425	0.392
5	0.571	0.325	0.194
6	0.434	0.497	0.299
7	0.442	0.459	0.395
8	0.009	0.726	0.156
9	0.441	0.635	0.088
10	0.593	0.134	−0.178
11	0.387	0.413	0.037
12	0.606	0.285	0.190
13	593.0	206.0	469.0
14	676.0	139.0-	236.0

Also, Table 5 presents the index of the model based on the Chi-square, Comparative Fit Index (CFI), Root Mean Square Error Approximation (RMSEA), the Goodness of Fit Index (GFI), and Adjusted Goodness of Fit Index (AGFI) values.

**Table 5.** Fit indices of VAT

**Chi-square**	**CFI**	**RMSEA**	**GFI**	**AGFI**
582.32	0.954	0.043	0.899	0.837

RMSEA is 0.043, a rate between 0 and 0.05, and CFI, GFI, and AGFI values are approximately 1, so the model has a good fitting.

## 4. Discussion

The Persian translation of the VAT is both reliable and valid, and suitable for clinical and research use in game addiction criteria with satisfactory properties. The findings of the present study should help future epidemiological studies on the occurrence and commonness of game addiction for Persian researchers. But, the limitations of using self-reporting scales should always be considered.

And according to the VAT questions, the critical factors found with factor analysis might be the negative reinforcement, then time, and eventually the control.

## Ethical Considerations

### Compliance with ethical guidelines

All ethical principles were considered in this article. The participants were informed about the purpose of the research and its implementation stages; they were also assured about the confidentiality of their information; Moreover, They were allowed to leave the study whenever they wish, and if desired, the results of the research would be available to them.
